# Pseudaminic Acid on *Campylobacter jejuni* Flagella Modulates Dendritic Cell IL-10 Expression via Siglec-10 Receptor: A Novel Flagellin-Host Interaction

**DOI:** 10.1093/infdis/jiu287

**Published:** 2014-05-13

**Authors:** Holly N. Stephenson, Dominic C. Mills, Hannah Jones, Enea Milioris, Alastair Copland, Nick Dorrell, Brendan W. Wren, Paul R. Crocker, David Escors, Mona Bajaj-Elliott

**Affiliations:** 1 Infectious Diseases and Microbiology Unit, Institute of Child Health, University College London; 2 Department of Pathogen Molecular Biology, London School of Hygiene and Tropical Medicine; 3 College of Life Sciences, University of Dundee; 4 Rayne Institute, University College London, United Kingdom; 5 Navarrabiomed-Fundacion Miguel Servet, Pamplona, Navarra, Spain

**Keywords:** *Campylobacter jejuni*, IL-10, dendritic cells, immune modulation, Siglec-10, p38

## Abstract

***Introduction.*** *Campylobacter jejuni* is a leading cause of bacterial gastroenteritis worldwide. At present the identity of host-pathogen interactions that promote successful bacterial colonisation remain ill defined. Herein, we aimed to investigate *C. jejuni*-mediated effects on dendritic cell (DC) immunity.

***Results.*** We found *C. jejuni* to be a potent inducer of human and murine DC interleukin 10 (IL-10) in vitro, a cellular event that was MyD88- and p38 MAPK-signalling dependent. Utilizing a series of *C. jejuni* isogenic mutants we found the major flagellin protein, FlaA, modulated IL-10 expression, an intriguing observation as *C. jejuni* FlaA is not a TLR5 agonist. Further analysis revealed pseudaminic acid residues on the flagella contributed to DC IL-10 expression. We identified the ability of both viable *C. jejuni* and purified flagellum to bind to Siglec-10, an immune-modulatory receptor. In vitro infection of Siglec-10 overexpressing cells resulted in increased IL-10 expression in a p38-dependent manner. Detection of Siglec-10 on intestinal CD11c^+^ CD103^+^ DCs added further credence to the notion that this novel interaction may contribute to immune outcome during human infection.

***Conclusions.*** We propose that unlike the *Salmonella* Typhimurium flagella-TLR5 driven pro-inflammatory axis, *C. jejuni* flagella instead promote an anti-inflammatory axis via glycan-Siglec-10 engagement.

*Campylobacter* species are a leading cause of bacterial gastroenteritis worldwide [1]. Evidence suggests that both the nature of the infecting strain and the host immune status defines the clinical presentation of human campylobacteriosis [1, 2]. Infection can result in asymptomatic carriage or clinical symptoms ranging from mild, watery, or bloody enteritis to autoimmunity [2, 3]. Research indicates that repeated exposure to different *C. jejuni* strains allows development of protective immunity that minimizes symptomatic disease while having a limited effect on colonisation [4, 5].

To promote their fitness, survival, and dissemination, enteropathogens utilize various strategies to counteract host immunity. For example, *C. jejuni* flagella evade Toll-like receptor (TLR)-5 recognition [6]. Pathogens are also known to target host anti-inflammatory cytokine interleukin 10 (IL-10) axis, for example, via TLR-2 engagement by *Yersinia sp.* V-antigen [7, 8], and via DC-SIGN engagement by *Mycobacterium tuberculosis* cell wall component, ManLAM [9]. IL-10 induction is potentially beneficial to pathogens as it can aid colonisation *via* immune suppression [10, 11]. C57BL/6 wild-type and IL-10 knockout mice are considered appropriate models for *C. jejuni* colonisation and colitis, respectively, suggesting that IL-10 signalling is a key determinant of clinical outcome to *C. jejuni* [12]. Although clearly important, currently there is limited information on how *C. jejuni* may modulate IL-10 immunity in the murine or the human host [13].

An emerging paradigm suggests that microbes can modulate host IL-10 production via engagement of glycan receptors [14]. Sialic-acid binding Ig-like lectins (Siglecs), I-type lectins, have emerged as important players in host immunity [15]. Siglecs bind sialylated structures, exhibiting varying specificities for the linkage of the sialic acid (Sia) and the underlying glycan structures that can be present both on host cells and microbes [15, 16]. The immunomodulatory capability of Siglecs occurs via the modulation of pattern-recognition receptor (PRR)-mediated signalling [17, 18]. Many sialylated pathogens engage with Siglecs, for example, the Sia moiety of different *C. jejuni* lipooligosaccharides (LOS) interacts with Siglec-7 and sialoadhesin (Siglec-1) [19–22].

The flagellin proteins of *C. jejuni* are *O*-linked glycosylated with Sia-like structures, derivatives of pseudaminic acid (Pse), and sometimes legionaminic acid (Leg), moieties important for flagella assembly and colonisation of chickens [23, 24]. Herein we report that derivatives of Pse on *C. jejuni* flagella interact with host Siglec-10 to modulate MyD88-mediated IL-10 expression *via* a p38-dependent pathway. The present study suggests *C. jejuni* flagella may favour interaction with immunomodulatory Siglec-10, an anti-inflammatory strategy that may promote asymptomatic colonisation in individuals that are repeatedly exposed to *Campylobacter sp.*

## MATERIALS AND METHODS

### Ethics Statement

Ethical approval for obtaining mucosal biopsies during endoscopy was granted by the Institute of Child Health/Great Ormond Street Hospital (ICH/GOSH) Research Ethics Committee (12/LO/0503 R&D 09GA03). Written informed consent was provided by the legal guardians of the study participants. Blood sampling from healthy volunteers was approved by ICH Human Tissue Act Review Board. Written informed consent was recorded in accordance with ICH Research Governance and Ethical Regulations.

C57BL/6 wild-type (WT) mice were purchased from the Jackson Laboratory (Maine, US). TRIF and MyD88 deficient murine bone-marrow were kindly provided by Prof Reis e Sousa (Cancer Research, UK). Ethical approval was obtained from University College London Ethics Committee (project license: PPL 80/2309).

### *C. jejuni* Culture

*C. jejuni* strains were routinely cultured on blood agar (BA) plates supplemented with *Campylobacter* selective supplement (Oxoid) and 7% (v/v) horse blood (TCS Microbiology) under microaerobic conditions at 37°C for 24 hours. The hypermotile strain 11168H is a variant of NCTC11168 WT [25]; 81-176 is a milk-borne outbreak isolate [26]. 11168H *flaA, pglB, waaF, kpsM, rpoN, Cj1324,* and *Cj1316* mutants and an 81-176 *flaA* mutant were obtained from the London School of Hygiene and Tropical Medicine *Campylobacter* Resource Facility (http://crf.lshtm.ac.uk/index.htm).

### Generation of Murine Bone-Marrow Derived Dendritic Cells (BMDCs)

The bone marrow from femurs and tibias of 6–12 week old C57BL/6 mice was extracted and the red blood cells lysed in Red Blood Cell Lysis Buffer (Sigma). Cells were seeded at 0.5 × 10^6^ cells/mL in IMDM containing 10% heat-inactivated fetal calf serum (FCS), 50 U/mL penicillin, 50 µg/mL streptomycin, 2 mM L-glutamine, 50 µM β-Mercaptoethanol, 10 µg/mL gentamicin, and 20 ng/mL murine GM-CSF (Invitrogen). On days 3 or 4, nonadherent cells were resuspended in fresh media containing GM-CSF. Cells were used on days 7 or 8. BMDCs were >80% positive for CD11c by flow cytometry.

### Generation of Human Monocyte-derived Dendritic Cells (hDCs)

CD14^+^ monocytes were extracted from peripheral blood using bead isolation (Miltenyi Biotec). In sum, 5 × 10^5^ cells/mL CD14^+^ monocytes were cultured in Roswell Park Memorial Institute medium (RPMI) containing 50 ng/mL interleukin 4 (IL-4) and 100 ng/mL GM-CSF (R&D systems) for 6–7 days. And hDCs were harvested and used immediately. Cells were >95% positive for CD11c by flow cytometry.

## RESULTS

### *C. jejuni* is a Potent Inducer of Innate IL-10 in Comparison to Other Enteropathogens

**Figure 1.**
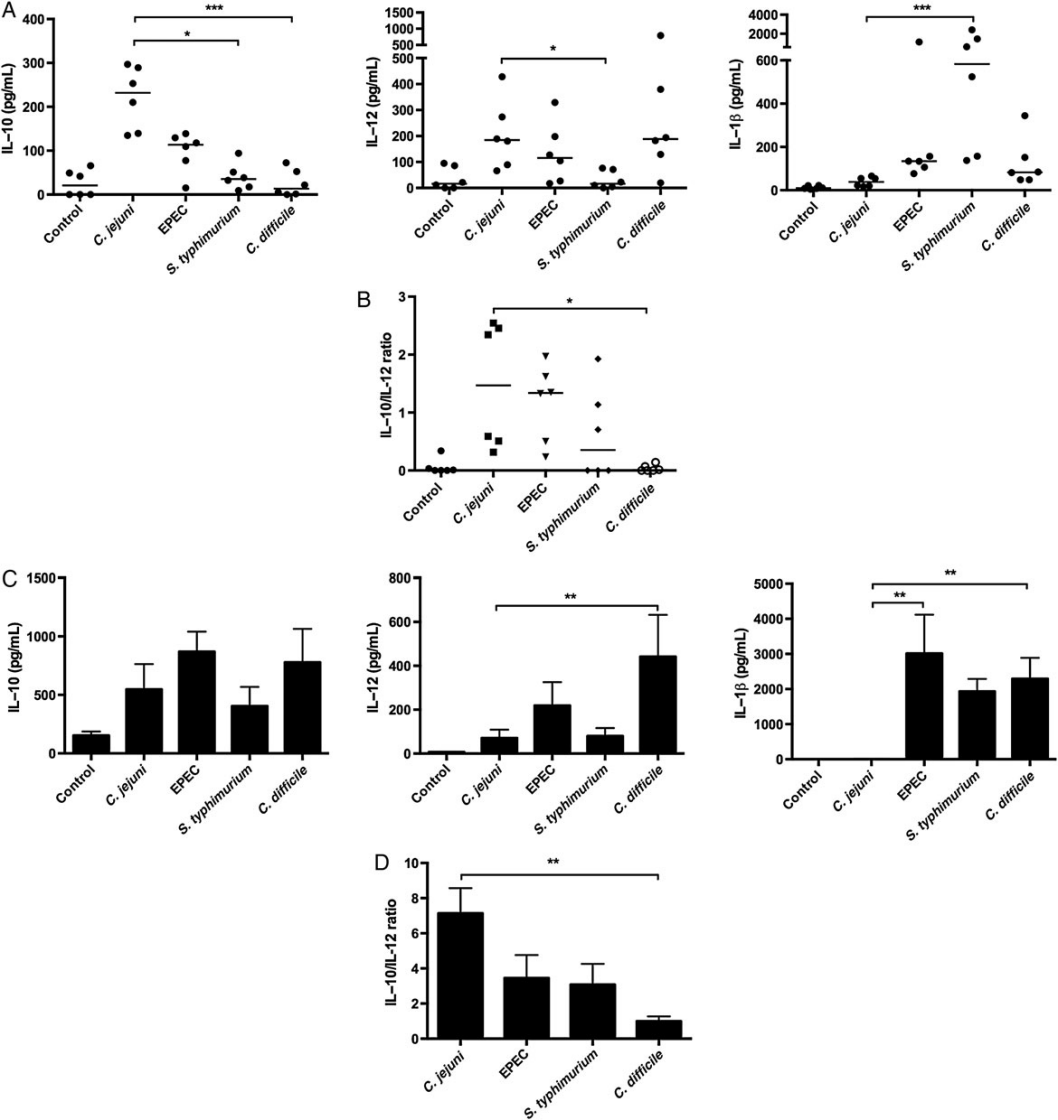
*Campylobacter jejuni* induces high IL-10 expression in hDC and murine BMDC. Cells were stimulated with either WT *C. jejuni* 11168H, EPEC E2348/69, *Salmonella* Typhiumurium SL1344, *Clostridium difficile* R20291 strains to reach a final MOI of 100, 8 hours post-infection. Cytokine levels were measured 8 hours post-infection. *A*, Enteropathogen-mediated hDC IL-10, IL-12 and IL-1β production. Bars depict median values from 6 individual donors. *B*, hDC IL-10/IL-12 ratio. Bars depict median values from 6 individual donors. *C*, Enteropathogen-mediated BMDC IL-10, IL-12 and IL-1β protein production. Bars show mean values from four independent experiments ± SEM (*D*) BMDC IL-10/IL-12 ratio. Bars show mean values from four independent experiments ± SEM. Friedman statistical analysis was performed on data sets. *P < .05; **P < .01; ***P < .001. Abbreviations: BMDC, bone-marrow derived dendritic cells; hDC, human dendritic cells; IL, interleukin; WT, wild-type.

Dendritic cells (DC) detect and respond to microbes via expression of costimulatory molecules and by generating a cytokine milieu, two cellular events that define adaptive immune outcome to infection. DC IL-10/IL-12 axis is a central determinant of T-cell immunity [27]. To define the DC cytokine milieu generated in response to *C. jejuni,* human (hDC) and bone-marrow derived (BMDC) DCs were employed. Studies were performed in vitro as the best murine model available is the antibiotic-treated IL-10 knock-out mouse, a model unsuitable for studying host anti-inflammatory immunity [28]. To improve our understanding of how microbes modulate host IL-10/IL-12 axis, 3 other well-studied enteropathogens *Enteropathogenic Escherichia coli EPEC* (E2348/69), *Salmonella* Typhimurium (SL1344), and *Clostridium difficile* (R20291) strains were also investigated. For appropriate comparison, infections were performed with live bacteria such that the final multiplicity of infection (MOI; hDCs and BMDCs) for each bacterial species reached 100 8 hours post-infection. Optimisation assays ensured low cell death during the experimental time-period (data not shown). In hDCs, *C. jejuni* induced the highest level of IL-10 (Figure 1*A*) while inducing comparable levels of IL-12 to *EPEC* and *C. difficile*. Interestingly, *C. jejuni* induced minimal levels of IL-1β (Figure 1*A*). A high IL-10/IL-12 ratio in response to *C. jejuni* was observed for 3/6 donors (Figure 1*B*). In BMDCs, *C. jejuni* induced comparable levels of IL-10 to the other enteropathogens (Figure 1*C*); however, lower levels of IL-12 in comparison to *EPEC* and *C. difficile* caused an overall high IL-10/IL-12 ratio (Figure 1*D*). As noted in hDCs, *C. jejuni* exhibited minimal capacity to elicit BMDC-derived IL-1β unlike the inflammasome-activating enteropathogens (Figure 1*C*). Taken together, the data suggested that *C. jejuni* is not a potent inducer of DC inflammasome activation but instead promotes IL-10 production.

### *C. jejuni* FlaA Modulates IL-10 Expression

**Figure 2.**
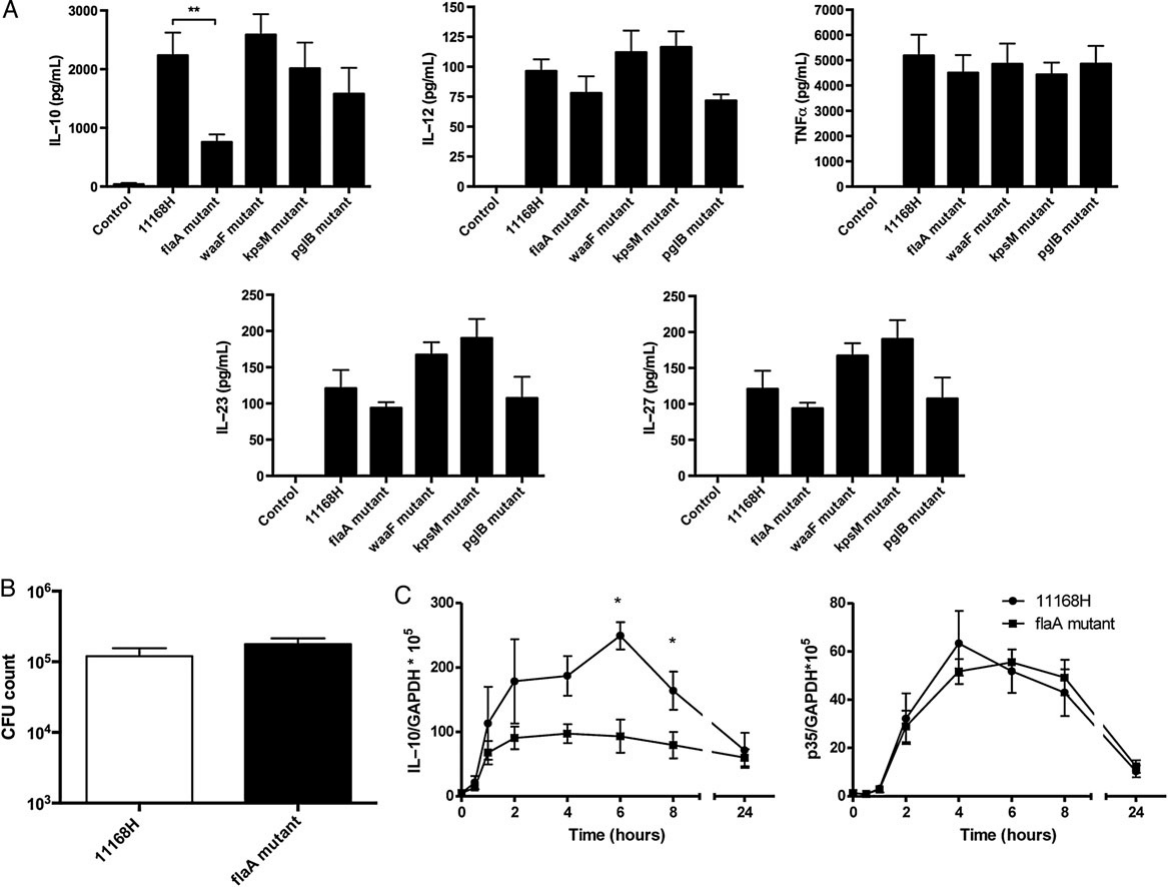
*Campylobacter jejuni* flagella selectively modulate BMDC IL-10 expression. *A*, BMDCs were stimulated with *C. jejuni* 11168H WT, *flaA*, *waaF, kpsM,* or *pglB* isogenic mutants for 24 hours MOI 100. Secreted IL-10, IL-12, TNFα, IL-23 and IL-27 cytokine levels were quantified by ELISA. Bars depict mean values ± SEM from four independent experiments. *B*, Uptake of 11168H WT and *flaA* isogenic mutant by BMDCs was quantified 4 hours post-infection (initial infection MOI 100) using the gentamicin protection assay. *C*, BMDCs were infected with *C. jejuni* 11168H WT or *flaA* isogenic mutant (MOI 100) and time-dependent IL-10 and IL-12 p35 subunit mRNA levels were quantified by real-time PCR. Bars depict mean values ± SEM from a minimum of 3 independent experiments. Friedman statistical analysis was performed on data sets. *P < .05; **P < .01; ***P < .001. Abbreviations: BMDC, bone-marrow derived dendritic cells; IL, interleukin; MOI, multiplicity of infection; PCR, polymerase chain reaction; SEM, standard error of the mean; TNFα, tumor necrosis factor α.

To identify *C. jejuni* structural components responsible for DC IL-10 expression, infection studies with *C. jejuni* 11168H WT and various mutants were performed (Figure 2*A*). A *C. jejuni flaA* mutant, which lacks the major flagellin protein FlaA but is still secretion-positive [29], showed significant reduction in IL-10 production compared to WT (Figure 2*A*; *P* < .01). Other structural mutants lacking the outer core oligosaccharide of the LOS (*waaF* [30]), capsular polysaccharide, CPS (*kpsM* [31]) or the *N*-linked glycosylation system (*pglB* [32]) showed no significant alteration in IL-10 protein induction. Interestingly, the effect of the *flaA* mutant was specific to IL-10 as no significant difference in the IL-12 family members (IL-12, IL-23 and IL-27) or TNF-α was observed between the WT and the mutants tested (Figure 2*A*). To confirm that the impact of *C. jejuni* flagella on IL-10 production was not due to a difference in BMDC uptake of the *flaA* mutant, a gentamicin protection assay was performed (Figure 2*B*). Similar numbers of intracellular bacteria were found between the WT and *flaA* mutant 4 hours post-infection, indicating low IL-10 expression in response to the *flaA* mutant was not due to defective phagocytosis.

The *C. jejuni* flagella-mediated effect on BMDC IL-10 expression was also noted at the transcriptional level (Figure 2*C*). Reduced IL-10 transcripts in response to infection with the *flaA* mutant were observed as early as 1 hour post-infection; this difference gained significance 6 hours post-infection (*P* < .05). Importantly, the effect on transcription was specific to IL-10 as IL-12 p35 subunit transcripts showed similar kinetics in response to the WT and the *flaA* mutant.

### Derivatives of Pseudaminic Acid (Pse) on *C. jejuni* FlaA Modulate BMDC and hDC IL-10 Production

**Figure 3.**
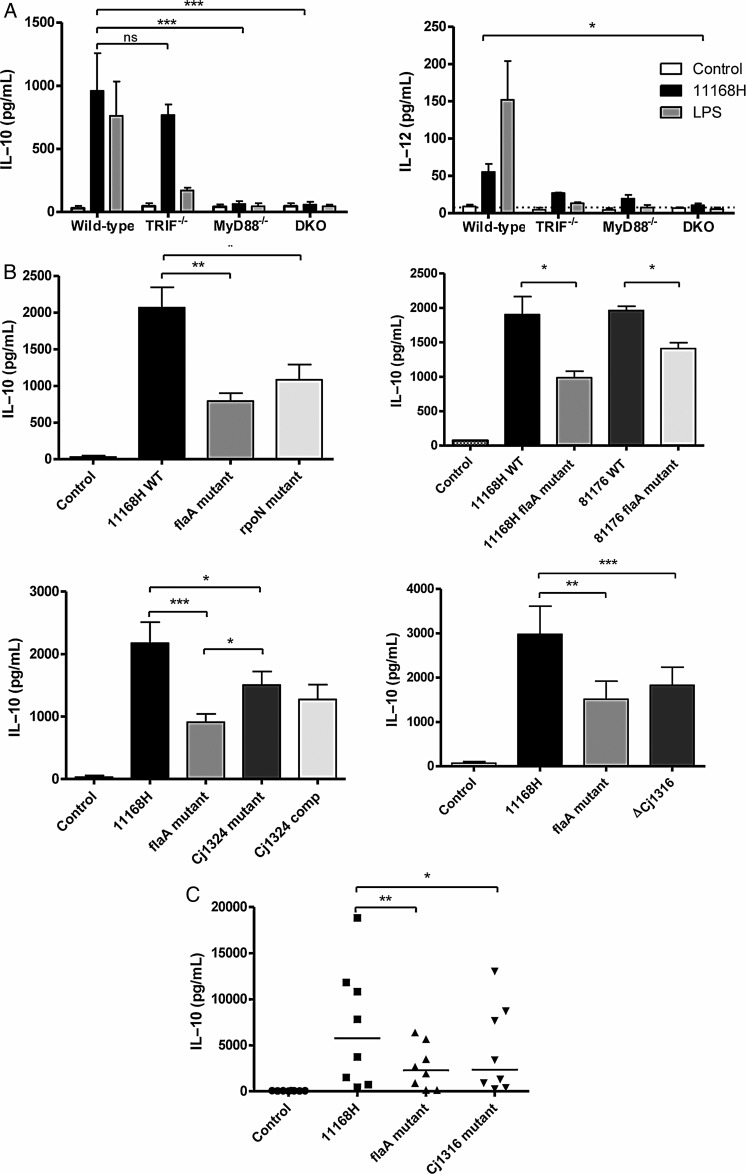
Pseudaminic acid structures on *Campylobacter jejuni* flagella modulate BMDC and hDC IL-10 expression. *A*, WT, TRIF^−/−^, MyD88^−/−^_,_ and DKO BMDCs were infected with 11168H WT *C. jejuni* or stimulated with LPS (100 ng/mL) for 24 hours. IL-10 and IL-12 protein levels post-stimulation were measured by ELISA. Bars depict mean levels ± SEM from 3 independent experiments. *B*, BMDCs were infected for 24 hours with 11168H WT, 11168H *flaA,* and 11168H *rpoN* mutants, 81-176 WT and 81-176 *flaA* mutant, 11168H *Cj1324* mutant, and the *Cj1324* complemented strain or 11168H *Cj1316* mutant (MOI 100). 24 hours post-infection IL-10 protein was measured by ELISA. Bars depict mean values ± SEM from four independent experiments. *C*, hDCs were infected with 11168H WT, *flaA,* and *Cj1316* mutant strains (MOI 100). Bars depict median levels from 8 individual donors. Friedman statistical analysis was performed on data sets. *P < .05; **P < .01; ***P < .001. Abbreviations: BMDC, bone-marrow derived dendritic cells; DKO, double knockout; ELISA, enzyme-linked immunosorbent assay; hDC, human dendritic cells; IL, interleukin; MOI, multiplicity of infection; SEM, standard error of the mean; WT, wild-type.

Toll-like receptor (TLR)-signalling is critical for *C. jejuni*-mediated BMDC cytokine production [33]. To determine the contribution of TLR-signalling on IL-10 expression, BMDCs from *Trif^−/−^, MyD88^−/−^,* and *Trif^−/−^ MyD88^−/−^* double knock-out (DKO) mice were generated. IL-10 expression was completely dependent on MyD88-mediated signalling (Figure 3*A*), whereas IL-12 was both TRIF and MyD88-dependent. As *C. jejuni* flagella do not interact with TLR5 [10], the bacterium instead must engage with other TLRs to elicit MyD88 dependent IL-10 expression. To determine which flagella component(s) modulate the observed TLR-MyD88 driven IL-10 expression, infection in the presence of a range of 11168H flagella-specific mutants was performed. A secretion-negative aflagellate *rpoN* mutant which lacks the alternative sigma factor σ^54^ [34] showed similar levels of IL-10 to the secretion-positive *flaA* mutant, suggesting that flagella-secreted proteins do not affect IL-10 (Figure 3*B*). Importantly, infection with an 81-176 *flaA* mutant showed comparable reduction in IL-10 when compared to its WT counterpart, suggesting this phenomenon was not specific to the 11168H WT strain (Figure 3*B*).

We next assessed the role of flagellin glycosylation in regulating IL-10 expression (Figure 3*B*). The Ptm and Pse pathways are necessary for the generation of Leg and Pse derivatives respectively in strain 11168H. The *Cj1324* mutant lacks 2 derivatives of Leg, Leg5AmNMe7Ac and Leg5Am7Ac, and in addition lacks a form of Pse, Pse5Ac7Am [23]. The *Cj1324* mutant induced significantly lower levels of IL-10 than the WT strain, although IL-10 levels were not restored to WT levels upon complementation (Figure 3*B*). It is important to note that the complemented mutant does not restore Pse5Ac7Am structures [23] which suggested that these structures may in fact be critical in modulating IL-10 expression. Strain 81-176 synthesizes only derivatives of Pse and not Leg supporting the notion that derivatives of Pse are important for the observed effect of *C. jejuni* flagella on hDC and BMDC IL-10. Mutations in the Pse biosynthesis pathway lead to defective flagellum assembly, due to the requirement of some but not all of the Pse modifications in filament polymerisation [24]. Mutation of *pseA* (*Cj1316*) results in flagellin monomers with only pse5Ac7Ac present and not pse5Ac7Am (or further derivatives), but the *Cj1316* mutant still have functional flagella [35, 36]. Infection with the *Cj1316* mutant showed a similar reduction in IL-10 secretion comparable to the *flaA* mutant (Figure 3*B*; *P* < .001). A total of 3 independently constructed *Cj1316* mutants were used in the assay and showed similar phenotypes (data not shown). Importantly, this phenotype was also observed in hDCs (Figure 3*C*; *P* < .05). This series of experiments suggest that the Pse5Ac7Am or further derivatives on *C. jejuni* flagella can modulate BMDC and hDC IL-10 production.

### *C. jejuni* Flagellin Pse Moieties’-host Siglec-10 Engagement Modulates DC IL-10

**Figure 4.**
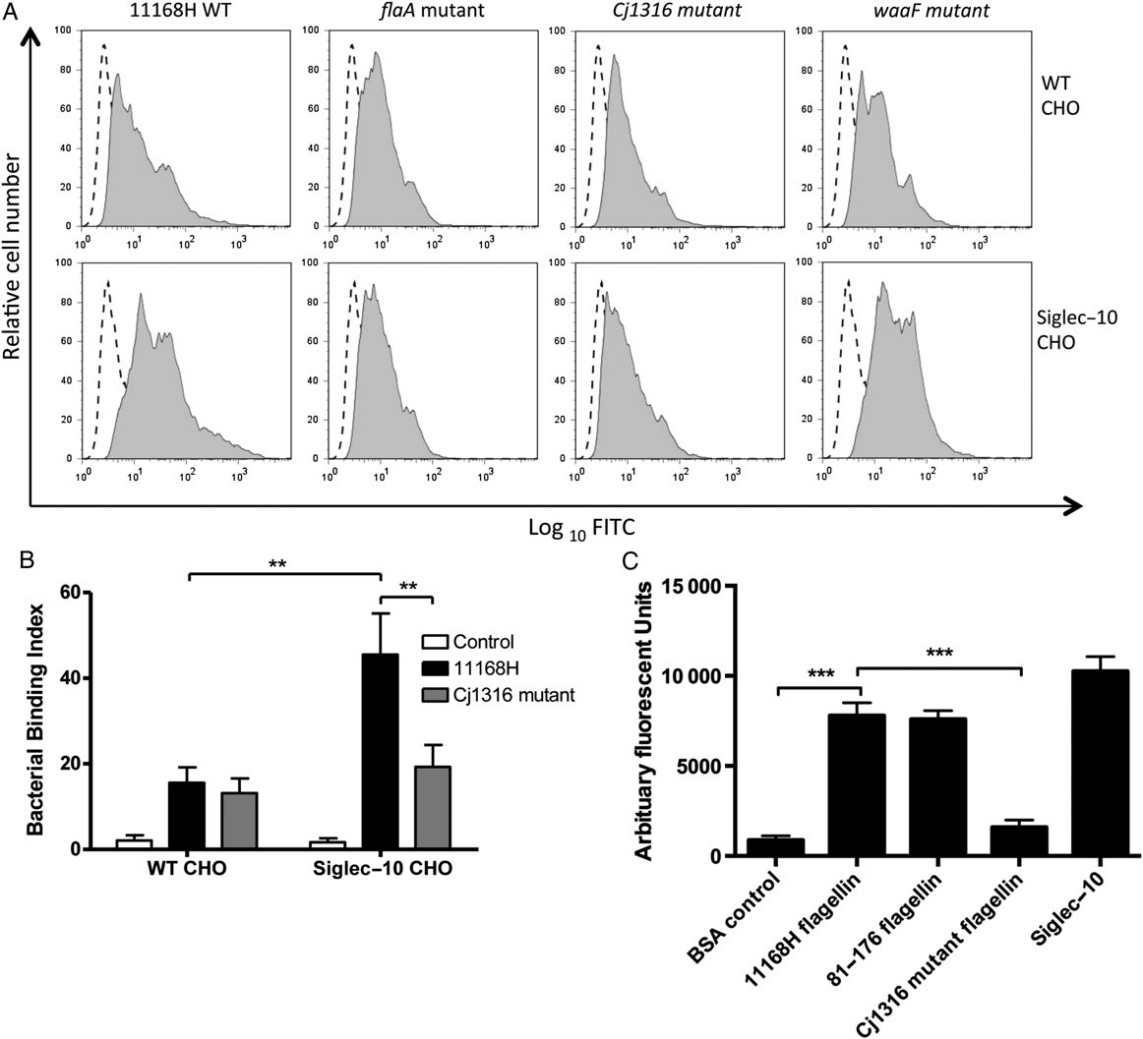
Glycosylated surface structures of *Campylobacter jejuni* flagella bind to Siglec-10 receptor. *A*, 11168H WT, *flaA, Cj1316,* and *waaF* mutants were assessed for binding to CHO cells overexpressing Siglec-10 receptor at 4°C. Histograms are representative of 3 independent experiments. Control cells alone (*dotted line*), bacteria/CHO cell co-cultures (*solid gray*). *B*, Binding of 11168H WT and *Cj1316* mutant to CHO cells is expressed as a bacterial binding index (% cells with bacteria bound × geometric mean fluorescence intensity of the bacteria-positive cells). Bars depict mean values ± SEM from 5 independent experiments. *C*, The binding capacity of Siglec-10-Fc to purified flagella from WT 11168H, WT 81-176 and 11168H *Cj1316* mutant was assessed by ELISA. Bars depict mean values ± SEM from 3 independent experiments performed in duplicate. Friedman statistical analysis was performed on data sets. *P < .05; **P < .01; ***P < .001. Abbreviations: CHO, Chinese hamster ovary; ELISA, enzyme-linked immunosorbent assay; IL, interleukin; MOI, multiplicity of infection; SEM, standard error of the mean; WT, wild-type.

A previous study highlighted a potential interaction between *C. jejuni* and Siglec-10, this interaction was found to be sialidase-insensitive indicating that the bacterial LOS is not the candidate ligand [19]. The core ring structure of Pse and Leg could potentially allow their accommodation in the Sia binding pocket of Siglecs [37]. This led us to hypothesize that derivative(s) of Pse may be potential ligands for Siglec-10 [19]. To test this hypothesis, the binding potential between live bacteria and Siglec-10-expressing Chinese hamster ovary (CHO) cells was investigated (Figure 4*A* and 4*B*). A significant increase in binding of *C. jejuni* WT to Siglec-10 expressing cells was observed. Importantly, this increase was not observed with the *flaA* and *Cj1316* mutants (*P* < .01). To confirm that the interaction was independent of the Sia on the bacterial LOS, the *waaF* mutant lacking the Sia residue on the OS outer core was employed (Figure 4*A*). The *waaF* mutant showed similar binding to Siglec-10 CHO cells to 11168H WT confirming that the oligosaccharide Sia moiety was not critical for this interaction.

To confirm the interaction between *C. jejuni* flagella and Siglec-10, flagella from 2 *C. jejuni* WT strains and from the *Cj1316* mutant was purified and coated onto high-absorbance plates, enzyme-linked immunosorbent assays (ELISAs) were performed with soluble Siglec-10-Fc to determine their binding potential (Figure 4*C*). Flagellin from both WT strains bound Siglec-10-Fc, whereas the flagellin from the 11168H *Cj1316* mutant failed to do so (*P* < .001). Collectively, our observations suggest for the first time to our knowledge that *C. jejuni* Pse modifications can actively engage with Siglec-10 receptor.

**Figure 5.**
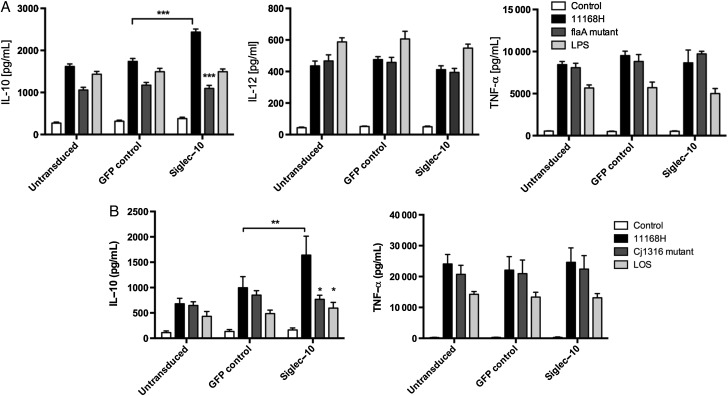
Siglec-10 receptor overexpression promotes IL-10 expression in response to *Campylobacter jejuni* flagella. *A*, Untransduced, GFP control, and Siglec-10 transduced PMA-differentiated THP-1 cells were infected with 11168H WT and *flaA* isogenic mutant (MOI 100) or LPS (100 ng/mL) for 24 hours. *B*, Untransduced, GFP control and Siglec-10 transduced RAW264.7 were infected with 11168H WT and *Cj1316* isogenic mutant (MOI 100) or purified *C. jejuni* 11168H LOS (100 ng/mL) for 24 hours. IL-10, IL-12 and TNFα protein levels were assessed by ELISA. Bars depict data from 4 independent experiments ± SEM. Friedman statistical analysis was performed on data sets. *P < .05; **P < .01; ***P < .001. Abbreviations: ELISA, enzyme-linked immunosorbent assay; IL, interleukin; MOI, multiplicity of infection; SEM, standard error of the mean; TNFα, tumor necrosis factor α; WT, wild-type.

To obtain direct evidence that *C. jejuni* interaction with the Siglec-10 promotes IL-10 production Siglec-10 was overexpressed in the human and murine macrophage cell lines THP-1 and RAW264.7, respectively, by lentiviral transduction. Overexpression was confirmed by flow cytometry (data not shown). In THP-1 cells *C. jejuni* 11168H WT infection led to an increase in IL-10 secretion from Siglec-10 transduced cells compared to GFP control-transduced cells (Figure 5*A*; *P* < .001). This increase was not observed upon infection with the 11168H *flaA* mutant (*P* < .001). *C. jejuni* 11168H WT infection led to approximately 2-fold increase in IL-10 secretion from Siglec-10 RAW264.7 transduced cells, compared to infection in GFP control-transduced cells (Figure 5*B*; *P* < .01). No increase was observed in response to the *Cj1316* mutant or to purified LOS (*P* < .05) suggesting specific engagement of Siglec-10 by *C. jejuni* flagella was responsible for the observed IL-10 response (*P* < .05). Interestingly, no significant increase in IL-12 (THP-1 cells) or tumor necrosis factor α (TNF-α; THP-1 and RAW264.7 cells) was observed in Siglec-10 overexpressing cells, indicating that WT *C. jejuni* mediates a specific effect on IL-10 in response to Siglec-10 engagement (Figure 5).

### MAPK Signalling Mediates Siglec-10 Induced IL-10 Expression

**Figure 6.**
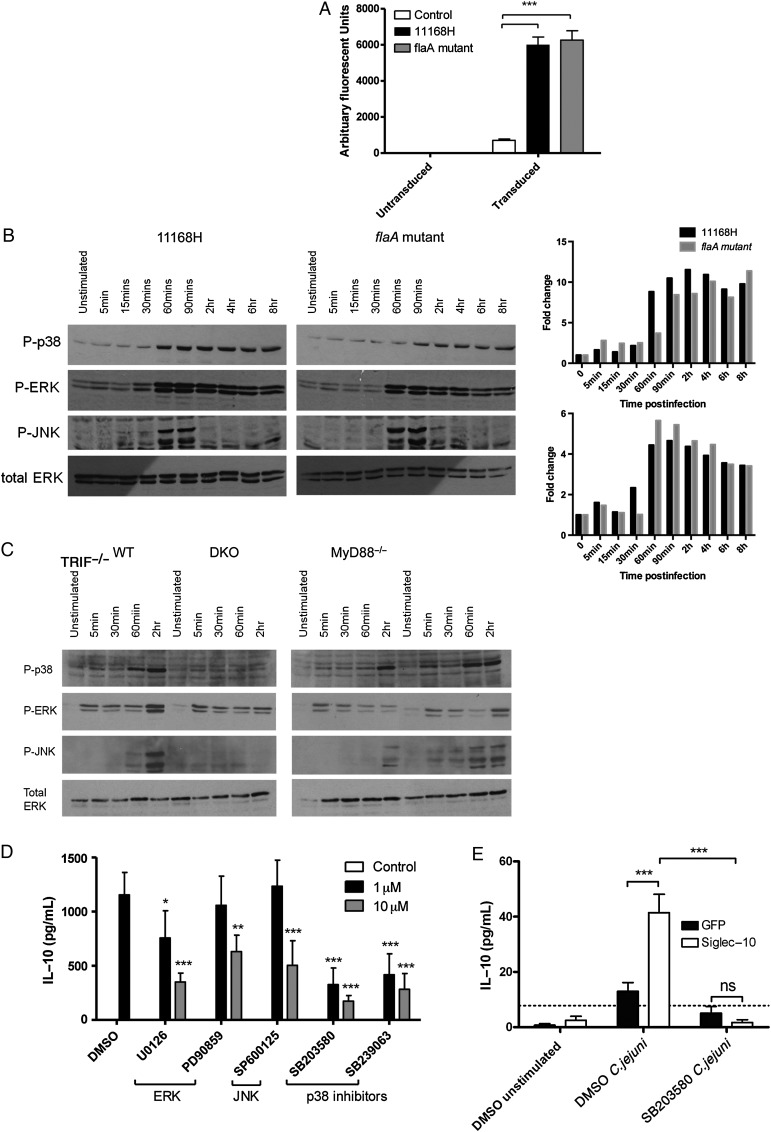
*Campylobacter jejuni* flagella alter BMDC IL-10 transcription in an NF-κB independent but MAPK-dependent manner. *A*, BMDCs were transduced with an NF-κB luciferase reporter plasmid on day 4 of differentiation. On day 8 BMDCs were infected with 11168H WT or *flaA* mutant (MOI of 100) for 6 hours and subsequent luciferase activity quantified. Bars depict values from 3 independent experiments performed in duplicate ± SEM. *B*, BMDCs were infected with 11168H WT or *flaA* isogenic mutant (MOI 100) and time-dependent effects on MAPK activation (phosphorylated p38, ERK and JNK) was followed by Western blotting; total ERK was used as a loading control. Representative blots from 3 independent experiments are shown. Densitometric analysis was performed on P-p38 and P-ERK blots using ImageJ software. *C*, WT, DKO, MyD88^−/−^ and TRIF^−/−^ BMDCs were infected with *C. jejuni* 11168H WT (MOI 100)*.* Time-dependent activation of MAPK pathways (p38, ERK, and JNK) was followed by Western blotting. The presence of phosphorylated MAPKs was indicative of activation; total ERK served as a loading control. *D*, BMDCs were pre-treated with various MAPK inhibitors for 2 hours and subsequently infected with *C. jejuni* 11168H WT (MOI 100). 1 μM (*black bars*; 5 μM PD90859) and 10 μM (*gray bars*; 50 μM PD90859). IL-10 and IL-12 protein levels were quantified 24 hours post-infection. *E*, Siglec-10 and GFP control transduced RAW264.7 cells were infected with 11168H WT *C. jejuni* (MOI of 100) for 24 hours in the presence of the p38 inhibitor SB203580 or DMSO vehicle control. IL-10 levels were analysed by ELISA. Blots are representative of 3 independent experiments. Friedman statistical analysis was performed on data sets. *P < .05; **P < .01; ***P < .001. Abbreviations: BMDC, bone-marrow derived dendritic cells; DKO, double knockout; ELISA, enzyme-linked immunosorbent assay; ERK, extracellular signal-regulated kinase; IL, interleukin; JNK, c-Jun N-terminal kinase; MAPK, mitogen-activated protein kinase; MOI, multiplicity of infection; SEM, standard error of the mean; WT, wild-type.

The transcription factor NF-κB and the mitogen-activated protein kinase (MAPK) pathways are central regulators of innate cytokine production. To assess whether NF-κB signalling was altered in response to the *flaA* mutant, BMDCs were transduced with an NF-κB responsive promoter that drives luciferase expression. The *flaA* mutant elicited NF-κB activation to a similar extent as the WT strain (Figure 6*A*). Analysis of the MAPK signalling, however, revealed a clear difference in the kinetics of p38 activation (Figure 6*B*). *C. jejuni* 11168H WT mediated marked p38 phosphorylation; in contrast the *flaA* mutant elicited slower kinetics with no apparent increase 60 minutes postinfection. Modest reduction in extracellular signal-regulated kinase (ERK) activation 30 minutes post-infection was also noted between the *flaA* mutant and the WT. In contrast, c-Jun N-terminal kinase (JNK) activation was similar in response to both WT and mutant. Taken together, the data indicated that *C. jejuni* flagella could potentially modulate DC IL-10 expression *via* modulation of p38 activation. Interestingly, a similar delay in p38 activation paralleled reduced IL-10 production in *MyD88^−/−^* BMDCs infected with the WT strain (Figure 6*C*), Collectively, the results raise the hypothesis that flagella-DC interactions potentially converge with the TLR-MyD88 signalling cascade at p38 to modulate downstream IL-10 gene expression.

Chemical inhibition confirmed a key role for the p38 pathway as p38 inhibitors (SB203580 and SB239063) at 1 µM (low concentration) caused significant reduction in IL-10 protein (Figure 6*D*). At 10 µM (50 µM PD90859) all MAPK inhibitors tested impacted on IL-10 (Figure 6*D*). To assess the contribution of p38 signalling in response to Siglec-10 engagement, infection in the presence of SB203580 was performed (Figure 6*E*). The increase in IL-10 secretion in the Siglec-10 overexpressing cells was abolished in the presence of the p38 inhibitor SB203580, confirming a critical role for p38-signalling in Siglec-10-dependent IL-10 secretion (*P* < .001). Overall, the evidence supports the notion that *C. jejuni* flagellin Pse-hDC Siglec-10 axis modulates IL-10 via a p38 MAPK-dependent mechanism(s).

### Siglec-10 is Expressed on Lamina Propria CD103+ Dendritic Cells

**Figure 7.**
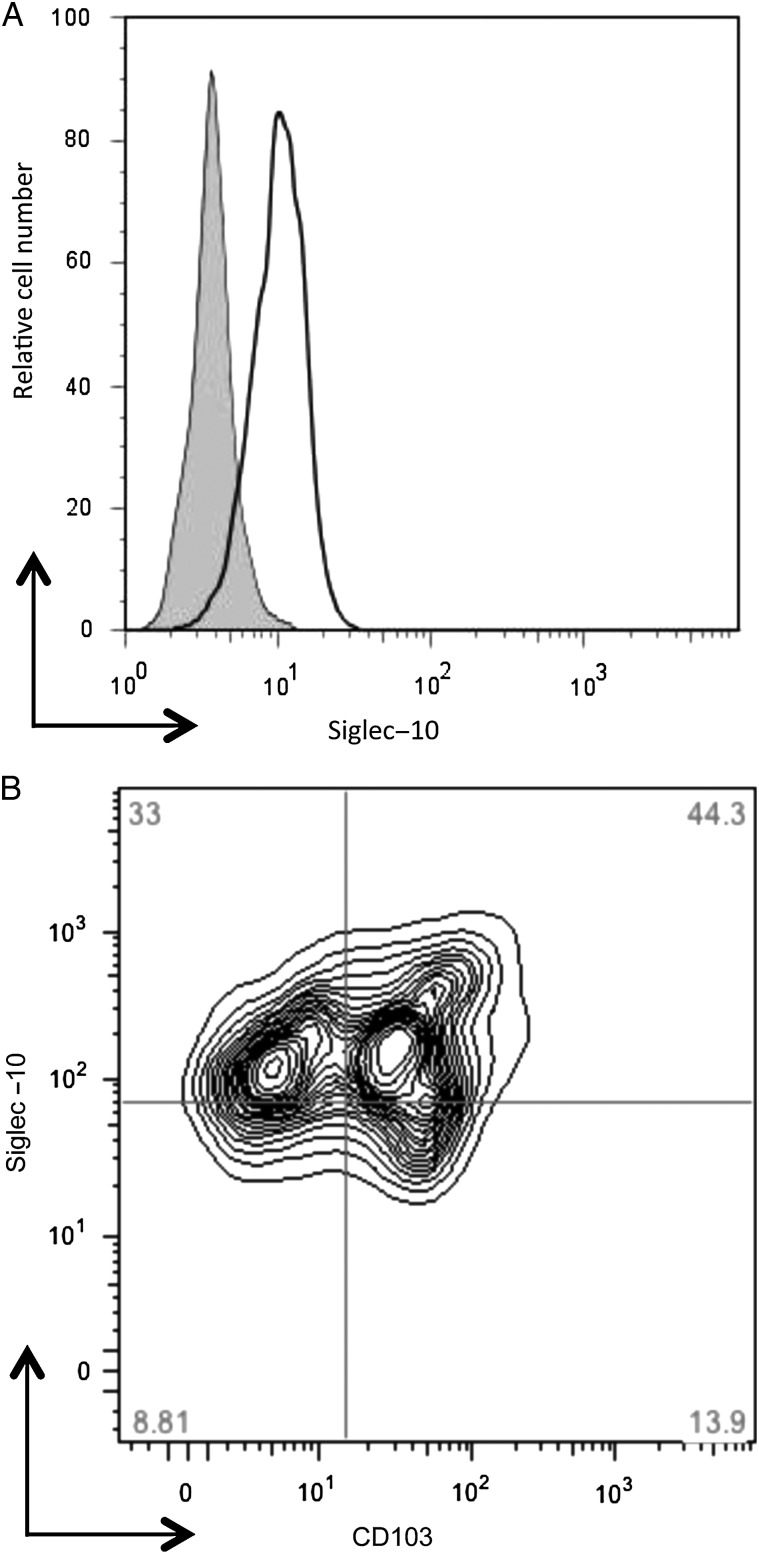
Siglec-10 is expressed on human peripheral blood monocyte-derived and gastrointestinal lamina propria dendritic cells. *A*, Human peripheral blood monocyte-derived DCs were stained with primary mouse monoclonal anti-Siglec-10, 5G6, and PE-conjugated donkey anti-mouse secondary antibody (*black line*), or secondary antibody alone (*solid gray line*). The histogram shown is from 1 representative donor of 4. *B*, Lamina propria cells from colonic biopsy tissue were analysed for expression of Siglec-10, CD103, and CD11c. CD11c^+^ cells were gated on and expression of CD103 and Siglec-10 assessed. FACS plots shown are from 1 representative donor of 3. Abbreviations: DC, dendritic cell; FACS, fluorescence activated cell sorting.

If this novel *C. jejuni*-host interaction does indeed contribute to immune outcome in humans, it was crucial to determine Siglec-10 expression on in vitro*-*cultured DCs and ex vivo human intestinal lamina propria (LP) DCs (Figure 7). In addition, expression of Siglec-G (murine homologue of Siglec-10) on BMDCs was also sought. Siglec-G showed strong intracellular staining on BMDCs (Supplementary Figure 1). In comparison, Siglec-10 was expressed on the surface of hDCs from 4 donors (Figure 7*A*). Siglec-10 surface staining was performed on colonic LP cells. CD11c^+^ cells were gated on to identify LP DCs. Cells were further separated by CD103 to define negative and positive populations for this molecule. Interestingly, the Siglec-10 staining was high in the CD11c^+^ CD103^+^ lamina propria DC population (Figure 7*B*), indicating that this receptor may indeed contribute to mucosal immunity to *C. jejuni*.

## DISCUSSION

The IL-10 axis is pivotal in defining the outcome of intestinal infection. The importance of this axis is demonstrated by the finding that IL-10 knock-out but not wild-type mice present with intestinal pathology in response to *C. jejuni* infection [12, 28].

Herein, we assign a potential novel function to *O*-linked glycosylated pseudaminic acid (Pse) structures on *C. jejuni* flagella in modulating innate DC IL-10 production, which indicates *C. jejuni* may have developed specific-mechanisms that promote anti-inflammatory immunity. The acute inflammation seen in the majority of naive individuals infected with *C. jejuni* suggests this anti-inflammatory mechanism may be more critical in asymptomatic infection that can occur after repeated exposure to *C. jejuni,* as highlighted in developing countries and long-term abattoir workers [5, 38–40].

Use of *C. jejuni* flagella glycosylation mutants implied that the carbohydrate modifications of FlaA were involved in modulating DC IL-10 expression. In particular, the Pse5Ac7Am moiety or further derivatives appeared to be critical. All *C. jejuni* strains studied express either Pse with its derivatives (eg, strain 81-176) or both Pse and Leg (eg, strain 11168H) [38]. 3 of the 19 *O*-linked glycosylated residues in strain 81-176 are critical for the assembly of functional flagella, 5 residues are important for auto-agglutination, and 11 residues have no ascribed function [24]. Their highly abundant nature on the flagellum surface suggests that these modifications may interact with the host. Mutation of the flagella Leg structures in strain 11168H decreases the ability of *C. jejuni* to colonize the chicken gastrointestinal (GI) tract confirming potential bacterial glycan-host crosstalk [23].

Siglecs are generally immunomodulatory [15, 16]. Pathogens can engage with host Siglecs promoting immune evasion, including the induction of IL-10 [41]. We defined the ability of both viable *C. jejuni* bacteria and purified flagella to bind to the Siglec-10 via Pse5Ac7Am or further derivatives. Although Sias are the best characterized ligands for Siglecs, these receptors are also able to bind other ligands [42, 43]. 9-carbon Pse shares many of the reported critical residues required for direct binding with the Siglec Sia binding pocket [37]. The carboxylate group, hydroxyl groups on the 4th and 8th carbon, and amide group on the 5th carbon are all conserved between pseudaminic acid and N-acetyl neuraminic acid (Sia). As yet, the structures of Siglec-G (murine homologue of Siglec 10) and Siglec-10 are unknown; the potential binding of Pse5Ac7Am to these receptors is therefore a matter for future research.

Siglecs can modulate macrophage IL-10 production following exposure to TLR ligands. Overexpression of Siglec-9 and Siglec-5 followed by TLR stimulation results in increased IL-10 [41]. Here, Siglec-10 was identified as the immunomodulatory receptor involved in *C. jejuni*-TLR mediated IL-10 responses. Utilizing chemical inhibitors and Siglec-10 overexpressing murine RAW264.7 cells we provide evidence that the bacterial Pse-host Siglec-10 interaction targets the p38 MAPK pathway for IL-10 modulation. Signalling events linking Siglec-10 engagement and p38 activation are currently unknown. Siglec-10 in addition to the intracellular immunoreceptor tyrosine-based inhibition motif (ITIM) domain also contains a putative growth factor receptor binding protein-2 (Grb2) binding motif [44], and interestingly Grb2-mediated signalling is known to alter p38 signalling [45]. How *C. jejuni*-mediated Siglec-10 engagement enhances p38 activity is currently under investigation.

Siglec-10-mediated IL-10 expression was dependent on Pse residues on *C. jejuni* flagella. For *C. jejuni* the main evolutionary driving forces may come from its interaction with the avian gut, in which it is a commensal. It is interesting to speculate how the abundant Sia and Sia-like *C. jejuni* surface structures may modulate avian immunity allowing commensalism to occur. The presence of Siglec-10 on human GI mucosal CD11c^+^ CD103^+^ DCs suggests that Siglec-10 may play an immunomodulatory role not only during *C. jejuni* infection but potentially also under other conditions, as CD103^+^ DCs exert tolerogenic functions [46, 47]. Interestingly, LP CD103^+^ DCs are known to induce regulatory T-cell differentiation and activation [48]. It is tempting to speculate whether potential differences in these DC and other lamina propria cell subsets may be one likely explanation as to why humans and not mice present with acute inflammation in response to *C. jejuni* infection, and whether repeated infection with *C. jejuni* in humans leads to changes in mucosal cell populations that may lead to asymptomatic colonisation. Future studies will determine if Siglec 10-driven IL-10 expression presents a novel therapeutic target for GI inflammatory conditions.

*S.* Typhimurium flagella, a TLR5 agonist, promote inflammation by negative regulation of LPS-mediated IL-10 [48]. Our findings highlight an opposing phenomenon whereby *C. jejuni* flagella engage in a TLR-5 independent, Siglec-10 receptor dependent manner leading to positive regulation of IL-10. Better understanding of how mucosal pathogens manipulate host TLR and glycan receptors to create a preferred niche for successful colonisation and/or cause GI disease may aid in the design of better future therapeutics.

## Supplementary Data

Supplementary materials are available at *The Journal of Infectious Diseases* online (http://jid.oxfordjournals.org). Supplementary materials consist of data provided by the author that are published to benefit the reader. The posted materials are not copyedited. The contents of all supplementary data are the sole responsibility of the authors. Questions or messages regarding errors should be addressed to the author.

Supplementary Data
