# Supplementary material for: Pseudaminic Acid on Campylobacter jejuni Flagella Modulates Dendritic Cell IL-10 Expression via Siglec-10 Receptor: A Novel Flagellin-Host Interaction
Source: J Infect Dis. 2014 May 13;210(9):1487–98. doi: 10.1093/infdis/jiu287 (PMC4195440; doi:10.1093/infdis/jiu287)
Supplement: Supplementary Data [file supp_210_9_1487__index.html]

Pseudaminic Acid on Campylobacter jejuni Flagella Modulates Dendritic Cell IL-10 Expression via Siglec-10 Receptor: A Novel Flagellin-Host Interaction — Pseudaminic Acid on Campylobacter jejuni Flagella Modulates Dendritic Cell IL-10 Expression via Siglec-10 Receptor: A Novel Flagellin-Host Interaction — Supplementary Data 

# Pseudaminic Acid on *Campylobacter jejuni* Flagella Modulates Dendritic Cell IL-10 Expression via Siglec-10 Receptor: A Novel Flagellin-Host Interaction

## Supplementary Data

Supplementary Data

**Files in this Data Supplement:**

- Supplementary Data - Doc file
