# Supplementary material for: Pseudaminic Acid on Campylobacter jejuni Flagella Modulates Dendritic Cell IL-10 Expression via Siglec-10 Receptor: A Novel Flagellin-Host Interaction
Source: J Infect Dis. 2014 May 13;210(9):1487–98. doi: 10.1093/infdis/jiu287 (PMC4195440; doi:10.1093/infdis/jiu287)
Supplement: Supplementary Data [file supp_jiu287_jiu287supp_data.doc]

**Supplementary Information**

**Materials & Methods**

**FITC labelled- *C. jejuni***

1 x 10^9^ CFU/mL were mixed 1:1 with saturated Fluorescein isothiocyanate (FITC; Sigma) and incubated in the dark at 37ºC for 1 h with gentle agitation. After incubation bacteria were washed three times before resuspension in PBS.

**Gentamicin Protection Assay**

2 x 10^5^/mL BMDCs (RPMI/ 2 mM L-glutamine/ 10% FCS) were infected at an MOI 100 for 4 h at 37^o^C. Post-infection, cells were washed twice with sterile PBS, followed by addition of RPMI containing 150 μg/mL gentamicin and incubation for 2 h to kill extracellular bacteria. Following washing, cells were lysed in 0.5 mL 0.1% Triton-X 100 (Sigma) in PBS for 5 min. Colony forming units (CFU) were quantified.

**Real-time PCR**

mRNA transcripts were quantified by real-time PCR. Reaction master mixes contained 10 μL SYBR Green (Invitrogen) and 5 pmol of forward and reverse primer. PCR was performed in duplicate in a Rotor-Gene 6000 machine (Qiagen) as follows: Initial denaturation 95ºC for 10 min (1 cycle); denaturation 95ºC for 15 sec, annealing 58ºC for 30 sec, extension 72ºC for 30 sec (40 cycles). Primers utilized: IL-10 fwd GGTTGCCAAGCCTTATCGGA, IL-10 rev ACCTGCTCCACTGCCTTGCT, p35 fwd CCTCAGTTTGGCCAGGGTC, p35 rev CAGGTTTCGGGACTGGCTAAG.

***C. jejuni*-mediated BMDC MAPK activation**

1 x 10^6^/mL BMDCs were cultured in RPMI containing 2 mM L-glutamine and 0.05% FCS, a day prior to infection with *C. jejuni* at an MOI 100. After inoculation, cells and bacteria were spun (1500 rpm, 5mins) to promote bacterial-cell association. Post-infection, cells were lysed in 50 μL lysis buffer [1% Triton-X 100 (Sigma), 150 mM sodium chloride, 50 mM Tris pH 8.0, complete-mini protease inhibitors (Roche, Sussex, UK), phosphatase inhibitor cocktail (Roche)]. Phospho-specific primary antibodies (Cell Signaling, MA) were used to detect MAPK activation by Western blotting.

**Siglec-CHO adhesion experiments**

1 x 10^6^/mL Siglec-10 expressing CHO cells were resuspended in F12 media containing 0.5% FCS. 300 μL of the re-suspension was co-cultured with FITC-labelled *C. jejuni* (MOI 100, 2 h, 4ºC to inhibit phagocytosis) with gentle rotation. After washing, cells were fixed in 4% paraformaldehyde (PFA). Bacterial adherence was assessed by flow cytometry using a FACScalibur (BD Biosciences).

**Flagella purification**

Flagella purification was performed as previously described (27). Presence and purity of the flagellin proteins were assessed by SDS-PAGE and Coomassie staining.

**Siglec-10 ELISA**

High-binding ELISA plates (Costar) were coated overnight at 37ºC with 0.5 µg purified *C. jejuni* flagella or its lipooligosacchride (LOS) moiety (28). Plates were washed three times in PBS/Tween (0.05%) and blocked in 1% bovine serum albumin for 2 h at RT. 1.5 µg/mL Siglec-Fc chimeras were mixed with alkaline phosphatase-conjugated anti-human IgG Ab (1:1000; Sigma) for 1 h with rotation. Complexes were added to wells and incubated at RT for 2 h. Plates were washed five times before 10µM fluorescein diphosphate (Sigma) solution was added for 30min at 37ºC, followed by fluorescence measurement (FLUOStar Optima, MBG Labtech).

**Siglec-10 overexpression in RAW264.7 cells**

Siglec-10 cDNA was ligated into an expression pDUAL lentivector plasmid; viral propagation was conducted in a HEK293T packaging cell-line (50). RAW264.7 cells were transduced (MOI 10) and expression assessed by flow cytometry. 1 x 10^6^ cells/mL cells were seeded in a 24-well plate prior to stimulation with *C. jejuni* strains at an MOI 100. Cytokines were assessed 24h post-infection by ELISA (eBioscience).

**Siglec-10 overexpressing THP-1 cells**

THP-1 cells were transduced as above. 2.5 x 10^5^ /ml differentiated THP-1 cells (monocytic THP-1 cells differentiated in the presence of 50ng/ml PMA for 24h followed by 24h in media alone) were infected with *C. jejuni* strains at an MOI of 100. Cytokines were assessed 24h post-infection by ELISA (eBioscience).

**Lentivector Transduction of NF-κB reporter plasmid**

On day 4 of differentiation, BMDCs were transduced with lenti-viruses (MOI 10) containing an NF-κB-luciferase reporter plasmid in the presence of 5 μg/mL protamine sulphate (Sigma). Cells were grown for an additional 4 days. 1 x 10^5^ transduced BMDCs (100μL RPMI,10% FCS) were stimulated with *C. jejuni* MOI 100 or purified *C. jejuni* LOS (100 ng/mL) (28) for 6 h. Cells were equilibrated to RT, 100 μL Bright-GloTM (Promega) reagent was added for 2 mins before quantification of luciferase activity.

**Human Gastrointestinal Siglec-10 expression**

Pinch colonic biopsies (from children with clinical diagnosis of cow’s milk allergy but no sign of active disease as shown by the lack of eosinophil infiltration) undergoing immuno-phenotyping were incubated in 30 mM EDTA/PBS for 30 min at 37ºC prior to centrifugation (300g, 10min). The cell pellet was resuspended in RPMI containing 10% FCS, 1% streptomycin/penicillin, 10 μg/mL gentamicin and 0.5 mg/mL collagenase II and incubated for 1 h at 37ºC. Cell suspension was filtered, washed with FACS buffer (PBS + 0.2% BSA + 0.02% sodium azide) and counted. Cells (50,000-1000, 000 per stain) were labelled [2 μg/ml of FITC-antihuman Siglec-10 (PE), CD103 (APC), and CD11c (FITC)] for 30 min on ice followed by washing with FACS buffer and fixation with 4% PFA prior to analysis (FACScalibur).

**Intracellular Siglec-G staining**

For intracellular staining, cells were fixed for 10 min with 4% PFA and then washed with permeabilisation buffer (PBS + 0.2% BSA + 0.05% sodium azide and 0.2% saponin). Cells were stained with Siglec G-APC antibody for 30 min at 4ºC, washed with permeabilisation buffer and fixed with Cellfix prior to analysis (FACScalibur).

**Statistics**

Data for experiments of >5 repeats which showed normal distribution (parametric data) when plotted on a box and whisker plot were subjected to paired t-test. Experiments of <5 repeats where normal distribution of the data points was unknown Wilcoxon matched pairs test was performed. For comparison of more than two groups in the same experiments Repeated Measures ANOVA was used for parametric data using the Tukey post test to compare all columns of data. For non-parametric data when comparing more than two groups in the same experiment Friedman test analysis was performed with Dunns post test to compare all columns of data. All statistical analysis was performed using GraphPad 5 software.

**Supplementary Figure 1: Intracellular expression of Siglec-G in murine bone-marrow-derived dendritic cells.**

BMDCs were stained for Siglec-G expression. Isotype control (dashed line); extracellular staining (grey line); intracellular staining (black line).


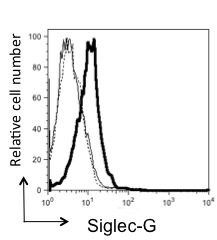


1 Power ME, Guerry P, McCubbin WD, Kay CM, Trust TJ. Structural and antigenic characteristics of Campylobacter coli FlaA flagellin. J Bacteriol **1994 Jun**;176(11):3303-13.

(2) Stephenson HN, John CM, Naz N, et al. Campylobacter jejuni lipooligosaccharide sialylation, phosphorylation and amide/ester linkage modifications fine-tune human Toll-like receptor 4 activation. J Biol Chem **2013 Apr 29**.

(3) Arce F, Breckpot K, Stephenson H, et al. Selective ERK activation differentiates mouse and human tolerogenic dendritic cells, expands antigen-specific regulatory T cells, and suppresses experimental inflammatory arthritis. Arthritis Rheum **2011 Jan**;63(1):84-95.
